# The Effect and Implication of Social Media Platforms on Cosmetic Facial Plastic Surgery Among Females in Saudi Arabia

**DOI:** 10.7759/cureus.60137

**Published:** 2024-05-12

**Authors:** Wafaa S Taishan, Mujtaba A Ali, Ibrahim Al Sulaiman, Kholoud Alsiwed, Assal Hobani, Ghada A Bin Abbas, Aseel AlOtaibi, Ahmed Aldhahri

**Affiliations:** 1 Department of Surgery, Faculty of Medicine, Al-Baha University, Al-Baha, SAU; 2 College of Medicine, Najran University, Najran, SAU; 3 College of Medicine, Fakeeh College for Medical Science, Jeddah, SAU; 4 College of Medicine, Ibn Sina Medical College, Jeddah, SAU; 5 College of Medicine, King Khaled University, Abha, SAU; 6 Faculty of Medicine, Majmaah University, Al Majma'ah, SAU; 7 College of Medicine, Ibn Sina National College, Jeddah, SAU

**Keywords:** perception of beauty, otolaryngology surgery, otolaryngology, facial surgery, cosmetic surgery, social media

## Abstract

Introduction

Facial plastic surgery addresses various facial imperfections, offering a range of procedures like rhinoplasty and facelifts. Social media promotes unrealistic beauty standards, leading to increased demand for such surgeries. Studies highlight its influence, emphasizing the need for research in this area. Our study aimed to evaluate the effect of social media advertisements and selfies on facial cosmetic surgery decisions and plans among females in Saudi Arabia.

Methodology

This is a cross-sectional study conducted in the Kingdom of Saudi Arabia that targeted females aged 18-80 years old. An electronic questionnaire in Arabic was used for data collection. Data was analyzed in IBM SPSS Statistics for Windows, Version 29 (Released 2023; IBM Corp., Armonk, New York, United States).

Results

Our study assessed 568 Saudi females regarding social media's impact on facial cosmetic surgery. Most of them were aged 21-30 years (39.4%) and Saudi nationals (94.2%). The majority, 87.9% (n=499), had not undergone cosmetic surgeries, and 12.1% (n=69) had; 68.1% (n=387) did not plan future surgeries. Notably, 42.6% (n=242) cited surgeon self-advertising and 38.0% (n=216) better selfies as an influencing factor in their cosmetic surgery decision. Logistic regression revealed several significant predictors of cosmetic surgery decisions including surgeon's advertisement (Exp(B) = 2.812, p < 0.001), cosmetic show viewing (Exp(B) = 2.327, p = 0.004), and social media photos (Exp(B) = 2.762, p = 0.001). Education (Exp(B) = 1.533, p = 0.035) and previous surgery (Exp(B) = 4.523, p < 0.001) correlated positively with considering surgery.

Conclusion

Our study highlights social media's influence on facial cosmetic surgery decisions among Saudi females. Surgeon advertisements, social media exposure, education, and previous surgery history emerged as significant predictors, warranting further research and targeted interventions.

## Introduction

Facial plastic surgery aims to enhance and improve the appearance of the face. It incorporates a variety of surgical techniques In order to treat issues including wrinkles, sagging skin, face asymmetry, and other defects [1,2]. This kind of surgery can improve a person's overall well-being and self-confidence by giving them a more young and refreshed appearance [3]. In general, it entails microvascular restoration of the head and neck, repair of facial deformities, blepharoplasty, facelifts, rhinoplasty, and browlifts [4].

The impact of social media on facial plastic surgery has been significant. With the rise of platforms like Instagram (Meta Platforms, Inc., Menlo Park, California, United States) and Snapchat (Snap Inc., Santa Monica, California, United States), people are increasingly exposed to filtered and edited images that promote unrealistic beauty standards [5,6]. This has led to a surge in individuals seeking facial plastic surgery procedures to achieve the flawless appearance they see online. Moreover, social media has also provided a platform for individuals to share their plastic surgery experiences, both positive and negative, which can influence others' decisions [7]. However, it is important to note that social media can also create a sense of pressure and insecurity, as individuals compare themselves to heavily edited images, leading to potential psychological implications [8].

A cross-sectional study was conducted in different regions of Saudi Arabia with 911 participants and found that most of them were influenced by the before-and-after cosmetic pictures and the desire to appear good in selfies and photographs, and, most importantly, all those who had undergone plastic surgery were affected by social media, surgeons' advertisements, and different TV programs [9]. Another study conducted in Riyadh among 816 female university students found that participants who viewed cosmetic surgery-related on social media, spent more time on social media, and had a negative self-image had a greater probability of considering plastic surgery in the future [10]. A cross-sectional study in Saudi Arabia from 2021 to 2022 among 2248 participants found that those who are affected by social media were more interested in doing cosmetic surgery compared to those who were not influenced, with Snapchat being the platform with the greatest influence [10,11].

There has been a significant rise in the demand for elective cosmetic surgery, and it is crucial to research the factors that might have influenced this rise in recent times. Studying the effect of social media and whether it plays a role in influencing the public to undergo cosmetic surgery might better explain the increase in demand for plastic surgery. Therefore, this study aims to assess the effect of social media, TV shows, and self-advertisements, determine the role of social media platforms in the decision-making process for facial cosmetic surgery, and evaluate the attitudes towards facial surgery among Saudi females.

## Materials and methods

This was a cross-sectional study conducted from March 10 to April 10, 2024, to evaluate the effect of social media, TV shows, and self-advertisements among females in different regions of Saudi Arabia, determine the role of social media platforms in the decision-making process for facial cosmetic surgery, and assess the attitude towards facial surgery among Saudi females. Both Saudi and non-Saudi females living in Saudi Arabia and aged between 18 and 80 years, who agreed to participate in the current survey, were included in the study.

The study was approved by the Institutional Research Board of Al-Baha University number (approval number: REC/SUR/BU-FM/2024/24). The participants were informed about the study aims and assured of data confidentiality, and consent was obtained from each participant before participating in the study.

Sample size

The sample size was calculated using Cochran’s equation with a precision level of ±5% and a confidence level of 95%. The estimated number of females in Saudi Arabia is about 15.96 million, and the calculated sample size was 385. The study enlisted 568 participants.

Data collection

An anonymous, self-administered validated electronic questionnaire was distributed through Telegram (Telegram Group Inc., Dubai, United Arab Emirates) and WhatsApp (Meta Platforms, Inc., Menlo Park, California, United States) among female residents of Saudi Arabia. The questionnaire was available in two languages, English and Arabic. The participants were free to choose the language they preferred. The questionnaire comprised two sections: Section A captured sociodemographic data, and Section B assessed the participants’ attitudes toward facial plastic cosmetic surgery. A pilot study was conducted on a small sample of 20 participants to test the suitability and clarity of the questionnaire and to estimate the time required for data collection 

Statistical analysis

A comprehensive statistical analysis was conducted on the dataset, encompassing both descriptive and inferential methodologies. Firstly, a descriptive analysis was conducted to summarize the demographic characteristics of the participants, which included age, gender, and other features. This provided an overview of the study population. Subsequently, inferential analyses such as the binary logistic regression model were employed to identify the predictors of decision-making in surgery. The Chi-square test is used to see the association between residency and the decision-making process. Statistical significance was established at a p-value of 0.05 or lower and a 95% confidence interval (CI). All statistical analyses are executed using IBM SPSS Statistics for Windows, Version 29.0 (Released 2023; IBM Corp., Armonk, New York, United States).

## Results

Our study included 568 Saudi females assessed for the influence of media platforms on facial plastic surgery decisions. As shown in Table 1, the majority of respondents were aged between 21-30 years (n=224, 39.4%), followed by 31-40 years (n=134, 23.6%). Saudi nationals comprised 94.2% (n=535) of the sample, with a minority being non-Saudi (n=33, 5.8%). Regarding marital status, 54.4% (n=309) were married, while 41.7% (n=237) were single. In terms of education, 74.6% (n=424) had a university degree and 20.1% (n=114) had completed high school. Regarding monthly income, 44.7% (n=254) earned less than 5000 Saudi Riyal (SAR), while 27.8% (n=158) earned 5000-10,000 SAR. Regarding residency, most participants belong to Najran (n=152, 26.8%), Jeddah (n=125, 22%), and Riyadh (n=96, 16.9%).

**Table 1:** Sociodemographic and other parameters of study participants assessed for the impact of media platforms on facial plastic surgery (N=568) SAR: Saudi Riyal

Parameters		Frequency (Percentage)
Age	< 20 Years	39 (6.9)
	21-30 Years	224 (39.4)
	31-40 Years	134 (23.6)
	41-50 Years	141 (24.8)
	> 50 Years	30 (5.3)
Nationality	Non-Saudi	33 (5.8)
	Saudi	535 (94.2)
Marital Status	Single	237 (41.7)
	Married	309 (54.4)
	Widowed/Divorced	22 (3.9)
Education	Able to Read/Write, Up to Middle School	30 (5.3)
	High School	114 (20.1)
	University Education	424 (74.6)
Monthly Incomes	< 5000 SAR	254 (44.7)
	5000-10,000 SAR	158 (27.8)
	10,000-15,000 SAR	93 (16.4)
	> 15,000 SAR	63 (11.1)
Residency	Najran	152 (26.8)
	Jeddah	125 (22.0)
	Riyadh	96 (16.9)
	Al-Baha	56 (9.9)
	Eastern Province	50 (8.8)
	Abha	28 (4.9)
	Other	61 (10.7)

**Figure 1:**
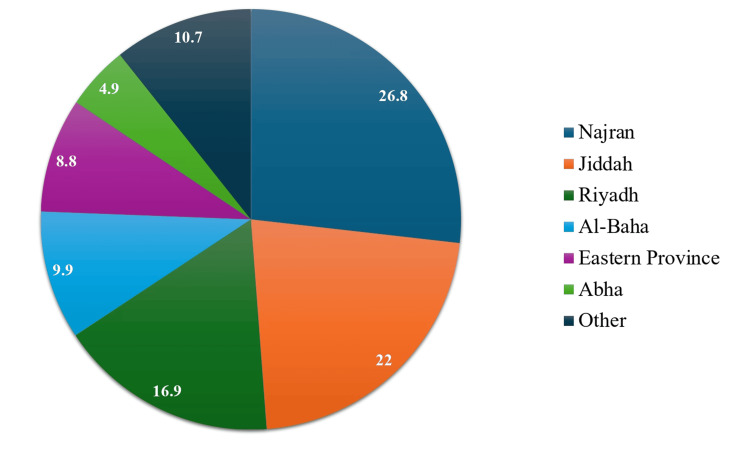
Distribution of study particpants among different cities or regions of KSA (N=568) KSA: Kingdom of Saudi Arabia

Figure 1 shows the distribution of females among different cities or regions of the Kingdom of Saudi Arabia (KSA). The largest proportion of females in the sample resided in Najran, accounting for 26.8%. Jeddah followed with 22%, while Riyadh comprised 16.9%. Al-Baha and the Eastern Province represented 9.9% and 8.8%, respectively. Abha constituted 4.9% of the sample, and the remaining areas made up 10.7%. 

Table 2 presents the descriptive statistics concerning Saudi female participants' attitudes toward facial surgery. The majority (87.9%, n=499) had not undergone any cosmetic interventions or plastic surgeries, while 12.1% (n=69) had. Regarding future plans, 68.1% (n=387) expressed no intention to undergo plastic surgery. Concerning selfie habits, a majority of respondents (61.8%, n=351) reported taking zero to one selfie daily. A significant proportion indicated that plastic surgeons' advertisements on various platforms influenced their decisions regarding plastic surgery (42.6%, n=242). Furthermore, watching cosmetic TV shows (32.4%, n=184) and viewing before-and-after photos on social media (42.6%, n=242) were cited as factors that encouraged consideration of plastic surgery. Additionally, 38.0% (n=216) stated that the desire to enhance their appearance in selfies and photos influenced their contemplation of plastic surgery.

**Table 2:** Assessment of attitudes towards facial surgery among responders (N=568)

Attitude-Related Question		Frequency (Percentage)
Have you undergone any cosmetic interventions or plastic surgeries?	No	499 (87.9)
	Yes	69 (12.1)
Are you planning to undergo any plastic surgery?	No	387 (68.1)
	Yes	181 (31.9)
How many selfies do you take daily?	0-1	351 (61.8)
	1-5	162 (28.5)
	5-8	31 (5.5)
	> 8	24 (4.2)
Has plastic surgeons' self-advertising of their work influenced your decision to undergo plastic surgery, or consider it?	No	326 (57.4)
	Yes	242 (42.6)
Has watching cosmetic TV shows influenced you to undergo plastic surgery, or consider it?	No	384 (67.6)
	Yes	184 (32.4)
Have before-and-after photos on social media influenced you to undergo plastic surgery, or consider one?	No	326 (57.4)
	Yes	242 (42.6)
Has the desire to look better in selfies and photos influenced you to undergo plastic surgery, or consider it?	No	352 (62.0)
	Yes	216 (38.0)

Social media factors influencing the decision-making process for facial cosmetic surgery

The number of selfies taken per day did not significantly influence the decision-making process (B = -0.099, p = 0.520, Exp(B) = 0.906, 95%CI [0.670, 1.224]) (Table 3). However, several other factors showed significant associations. The advertisement of plastic surgeons' work had a highly significant positive effect (B = 1.034, p < 0.001, Exp(B) = 2.812, 95%CI [1.656, 4.774]), suggesting that exposure to such advertisements increased the likelihood of considering facial cosmetic surgery nearly threefold. Similarly, watching cosmetic shows (B = 0.845, p = 0.004, Exp(B) = 2.327, 95%CI [1.314, 4.122]) and being influenced by before-and-after photos uploaded on social media (B = 1.016, p = 0.001, Exp(B) = 2.762, 95%CI [1.530, 4.985]) were significantly associated with an increased likelihood of considering cosmetic surgery. Moreover, the desire to look better in selfies and photos had the strongest effect (B = 1.097, p < 0.001, Exp(B) = 2.994, 95%CI [1.793, 5.001]), indicating that individuals who expressed this desire were nearly three times more likely to consider facial cosmetic surgery.

**Table 3:** Social media factors influencing the decision-making process for facial cosmetic surgery among responders (Logistic Regression Model) Exp(B): odds ratio, CI: confidence interval

Social Media Factors	B	Sig.	Exp(B)	95% CI	
				Lower	Upper
Number of Selfies Taken Per Day	-.099	.520	.906	.670	1.224
Advertisement of Plastic Surgeons of Their Work	1.034	.000*	2.812	1.656	4.774
Watching Cosmetic Shows	.845	.004*	2.327	1.314	4.122
Influence of Photos Before/After Uploaded on Social Media	1.016	.001*	2.762	1.530	4.985
Desired to Look Better in Selfies and Photos	1.097	.000*	2.994	1.793	5.001
Constant	-2.548	.000	.078		

Sociodemographic factors influencing the decision-making process for facial cosmetic surgery

Higher education level showed a significant positive association (B = 0.427, p = 0.035, Exp(B) = 1.533, 95%CI [1.031, 2.279]), indicating that individuals with higher education were 1.533 times more likely to consider facial cosmetic surgery compared to those with lower education levels (Table 4). Additionally, higher monthly income exhibited a marginally significant positive association (B = 0.196, p = 0.053, Exp(B) = 1.217, 95%CI [0.998, 1.484]), suggesting that individuals with higher incomes were slightly more likely to consider facial cosmetic surgery. Notably, previous cosmetic surgery had a highly significant positive association (B = 1.509, p < 0.001, Exp(B) = 4.523, 95%CI [2.596, 7.881]), indicating that individuals with a history of previous cosmetic surgery were significantly more likely to consider it again. However, age showed a non-significant negative association with the decision-making process (B = -0.179, p = 0.124, Exp(B) = 0.836, 95%CI [0.665, 1.051]), indicating that age alone did not significantly influence the likelihood of considering facial cosmetic surgery. Similarly, nationality (being Saudi) did not significantly affect the decision-making process (B = 0.466, p = 0.321, Exp(B) = 1.594, 95%CI [0.635, 4.002]), nor did marital status (being married) (B = 0.195, p = 0.330, Exp(B) = 1.215, 95%CI [0.821, 1.798]). 

**Table 4:** Sociodemographic factors influencing the decision-making process for facial cosmetic surgery among responders (Logistic Regression Model) Exp(B): odds ratio, CI: confidence interval

Sociodemographic Factors	B	Sig.	Exp(B)	95% CI	
				Lower	Upper
Age	-.179	.124	.836	.665	1.051
Nationality (Saudi)	.466	.321	1.594	.635	4.002
Marital Status (Married)	.195	.330	1.215	.821	1.798
Higher Education	.427	.035	1.533	1.031	2.279
Higher Monthly Income	.196	.053	1.217	.998	1.484
Previous Cosmetic Surgery	1.509	.000	4.523	2.596	7.881
Constant	-2.758	.000	.063		

Place of residence as a factor in the decision-making process for facial cosmetic surgery

Table 5 represents the potential association between the decision-making process for facial cosmetic surgery among Saudi females and their residence areas using Chi-square test. The highest proportion of respondents planning to have plastic surgery was observed in Abha (n=13, 46.4%) and Al-Baha (n=24, 42.9%), while Jeddah (n=31, 24.8%) and Eastern Province (n=13, 26%) had slightly lower proportions; p-value was 0.019, indicating a statistically significant association between places of residence and the decision-making process for facial cosmetic surgery among Saudi females.

**Table 5:** Association between decision-making process for facial cosmetic surgery among study participants and the place of residence P=0.019 (Chi-Square)

Area of Residence	Planning to have Plastic Surgery		Total, n (%)
	No, n (%)	Yes, n (%)	
Najran	109 (71.7)	43 (28.3)	152 (100.0)
Jeddah	94 (75.2)	31 (24.8)	125 (100.0)
Riyadh	56 (58.3)	40 (41.7)	96 (100.0)
Al-Baha	32 (57.1)	24 (42.9)	56 (100.0)
Eastern Province	37 (74.0)	13 (26.0)	50 (100.0)
Abha	15 (53.6)	13 (46.4)	28 (100.0)
Other	44 (72.1)	17 (27.9)	61 (100.0)

## Discussion

Facial plastic surgery enhances facial appearance, addressing imperfections like wrinkles and asymmetry. Kim et al. show that increasing plastic surgery rates suggest a growing desire for aesthetic enhancement and appearance improvement among individuals [12]. Social media fosters unrealistic beauty standards, driving demand for procedures. Similarly, Mavis et al. show that social media affects the self-esteem of individuals with regard to body image, body modification, and how they view themselves in society [13]. Studies in Saudi Arabia link social media exposure to increased interest in cosmetic surgery, especially among youth [14]. Our study aimed to investigate the influence of social media platforms on the decision-making process for facial cosmetic surgery among females in Saudi Arabia. 

Notably, the demographic profile of the participants reflects a diverse range of ages, with the predominant age group being 21-30 years, indicating the interest of the young population in cosmetic procedures. This aligns with the global trend of increasing interest in cosmetic procedures among younger individuals. Similarly, a study by Amiri et al. shows the trend of increasing acceptance of cosmetic procedures among young participants [15]. Moreover, the overwhelming majority of the present study being Saudi nationals highlights the relevance of the study within the local cultural context.

However, the attitudes towards facial surgery among Saudi females revealed several important findings. While the majority had not undergone any cosmetic interventions, a significant proportion expressed an interest in future procedures. This suggests a growing acceptance and willingness to consider cosmetic surgery within the Saudi population. In their study, Almajnoni et al. show that most participants accepted cosmetic surgery, but further nationwide research is needed to gauge broader attitudes toward aesthetic procedures in Saudi Arabia [16]. The influence of social media platforms, particularly through plastic surgeons' self-advertising, cosmetic TV shows, and before-and-after photos on social media, emerged as significant factors shaping attitudes towards facial surgery. These findings corroborate previous research highlighting the impact of social media on body image ideals and cosmetic surgery desires. Similarly, a study by Walker et al. found that viewing images of females who have undergone cosmetic enhancements affected young women's desire for cosmetic surgery, especially if they spent a significant amount of time on social media, followed many accounts, and were less satisfied with their appearance [17].

Moreover, the logistic regression analysis in the current study identified key social media factors influencing the decision-making process for facial cosmetic surgery. Exposure to plastic surgeons' advertisements, cosmetic TV shows, and before-and-after photos on social media significantly increased the likelihood of considering cosmetic surgery. Notably, the desire to look better in selfies and photos emerged as the strongest predictor, emphasizing the role of self-image concerns in driving cosmetic surgery decisions. These findings highlight the pervasive influence of social media on beauty standards and the acceptance of cosmetic procedures, particularly among younger demographics. Similarly, Aldosari et al. recognized the influence of surgeon self-promotion, cosmetic TV programs, social media before-and-after images, and the desire for enhanced selfies on the trends observed in cosmetic surgery [18]. Moreover, Shome et al., in an experimental study in India, revealed the negative impact of selfie culture on well-being and increased demand for cosmetic surgery and that urgent action is needed to mitigate harmful effects on youth [19]. Additionally, in a study by Fox et al., selfie takers expressed higher self-focus, more negative mood, and diminished self-esteem compared to those taking pictures of objects [20].

Moreover, sociodemographic factors such as age, nationality, and marital status showed mixed associations with the decision-making process for facial cosmetic surgery in the current study. While age and nationality did not significantly influence the likelihood of considering surgery, higher education levels and higher monthly income were positively associated with considering cosmetic procedures. These findings suggest that individuals with higher socioeconomic status and educational levels show a trend towards cosmetic enhancements, possibly due to greater access to information and resources. However, Ramandi et al. showed that several other factors are associated with growing interest in cosmetic procedures including older age, having children, higher household income, lower education, and greater acceptance of media images [21].

Thus, our study confirms social media's significant influence on cosmetic surgery attitudes, consistent with prior research. Higher education, income, and past surgery correlate with facial surgery consideration, mirroring existing literature.

Limitations

Despite its contributions, our study has some limitations. The cross-sectional design limits establishing causality, and the sample may not be fully representative of the Saudi population. Additionally, self-reported data are subject to recall and social desirability biases. Future research could employ longitudinal designs and larger, more diverse samples to further explore the complex interplay between social media, cultural norms, and cosmetic surgery decisions.

Implications and Future Directions

Social media's influence on cosmetic surgery decisions is crucial for healthcare providers, authorities, and educators. Healthcare professionals must understand patients' perceptions and expectations while authorities should implement responsible advertising practices. Educational initiatives promoting media literacy and healthy body image ideals can mitigate negative effects on self-esteem and body image.

## Conclusions

Our study highlights the significant influence of social media and other personal factors on the decision-making process for facial cosmetic surgery among Saudi females. Exposure to plastic surgeons' advertisements, cosmetic TV shows, and before-and-after photos on social media increased the likelihood of considering cosmetic surgery. Higher education level, higher income, and previous cosmetic surgery history were also associated with a greater inclination toward facial cosmetic procedures.

## References

[1] Devgan L, Singh P, Durairaj K (2019). Surgical cosmetic procedures of the face. Otolaryngol Clin North Am.

[2] Roh DS, Panayi AC, Bhasin S, Orgill DP, Sinha I (2019). Implications of Aging in Plastic Surgery. Plast Reconstr Surg Glob Open.

[3] Most SP, Alsarraf R, Larrabee WF Jr (2002). Outcomes of facial cosmetic procedures. Facial Plast Surg.

[4] Chuang J, Barnes C, Wong BJ (2016). Overview of facial plastic surgery and current developments. Surg J (N Y).

[5] Laughter MR, Anderson JB, Maymone MB, Kroumpouzos G (2023). Psychology of aesthetics: beauty, social media, and body dysmorphic disorder. Clin Dermatol.

[6] Wang JV, Rieder EA, Schoenberg E, Zachary CB, Saedi N (2020). Patient perception of beauty on social media: professional and bioethical obligations in esthetics. J Cosmet Dermatol.

[7] Obeid FM, Mortada H, Alsulaiman M, Faisal AlSwaji G (2022). The use of social media and its influence on undergoing rhinoplasty. Plast Reconstr Surg Glob Open.

[8] Habib A, Ali T, Nazir Z, Mahfooz A (2022). Snapchat filters changing young women's attitudes. Ann Med Surg (Lond).

[9] Suwayyid W, Alshbini M, Alotaibi S, Habib L, Alotaibi S, Aboaziz R, Alkubaisy Y (2020). The public view of media impact on seeking cosmetic surgeries in Saudi Arabia: A cross sectional study. Med Sci.

[10] Arab K, Barasain O, Altaweel A (2019). Influence of social media on the decision to undergo a cosmetic procedure. Plast Reconstr Surg Glob Open.

[11] AlBahlal A, Alosaimi N, Bawadood M, AlHarbi A, AlSubhi F (2023). The effect and implication of social media platforms on plastic cosmetic surgery: a cross-sectional study in Saudi Arabia from 2021 to 2022. Aesthet Surg J Open Forum.

[12] Kim YJ, Park JW, Kim JM (2013). The functionality of facial appearance and its importance to a korean population. Arch Plast Surg.

[13] Henriques M, Patnaik D (2021). Social media and its effects on beauty. Beauty - Cosmetic Science, Cultural Issues and Creative Developments.

[14] Ateq K, Alhajji M, Alhusseini N (2024). The association between use of social media and the development of body dysmorphic disorder and attitudes toward cosmetic surgeries: a national survey. Front Public Health.

[15] AlShamlan NA, AlOmar RS, Al-Sahow AZ (2022). Cosmetic surgeries and procedures among youth in Saudi Arabia: a cross-sectional study of undergraduate university students in the Eastern Province. Postgrad Med J.

[16] Almajnoni RS, Alharbi M, K Aljindan F (2023). Acceptance and attitude toward cosmetic surgeries in the western region of Saudi Arabia: a cross-sectional survey. Cureus.

[17] Walker CE, Krumhuber EG, Dayan S, Furnham A (2019). Effects of social media. Curr Psychol.

[18] Aldosari BF, Alkarzae M, Almuhaya R, Aldhahri R, Alrashid H (2019). Effect of media on facial plastic surgery in Saudi Arabia. Cureus.

[19] Shome D, Vadera S, Male SR, Kapoor R (2020). Does taking selfies lead to increased desire to undergo cosmetic surgery. J Cosmet Dermatol.

[20] Fox J, Vendemia MA, Smith MA, Brehm NR (2021). Effects of taking selfies on women's self-objectification, mood, self-esteem, and social aggression toward female peers. Body Image.

[21] Darzi Ramandi S, Irandoust K, Hashempour R, Talebianpour H, Yahyavi Dizaj J, Moghimi F, Kazemi-Karyani A (2022). Inequality in cosmetic services and surgery among Iranian households in 2019: a decomposition analysis. World J Plast Surg.

